# Preventive Effects of Heat-Killed *Enterococcus faecalis* Strain EC-12 on Mouse Intestinal Tumor Development

**DOI:** 10.3390/ijms18040826

**Published:** 2017-04-13

**Authors:** Shingo Miyamoto, Masami Komiya, Gen Fujii, Takahiro Hamoya, Ruri Nakanishi, Kyoko Fujimoto, Shuya Tamura, Yurie Kurokawa, Maiko Takahashi, Tetsuo Ijichi, Michihiro Mutoh

**Affiliations:** 1 Epidemiology and Prevention Division, Research Center for Cancer Prevention and Screening, National Cancer Center, 5-1-1 Tsukiji, Chuo-ku, Tokyo 104-0045, Japan; shinmiya@ncc.go.jp (S.M.); mkomiya@ncc.go.jp (M.K.); thamoya@ncc.go.jp (T.H.); rnakanis@ncc.go.jp (R.N.); shtamura@ncc.go.jp (S.T.); yurkurok@ncc.go.jp (Y.K.); maitakah@ncc.go.jp (M.T.); 2 Division of Carcinogenesis and Cancer Prevention, National Cancer Center Research Institute, 5-1-1 Tsukiji, Chuo-ku, Tokyo 104-0045, Japan; gfujii@ncc.go.jp; 3 Division of Molecular Biology, Nagasaki International University, 2825-7 Huis Ten Bosch, Sasebo, Nagasaki 859-3298, Japan; kfujit@niu.ac.jp; 4 Combi Corporation, Functional Foods Division, 5-2-39, Nishibori, Sakura-ku, Saitama-shi, Saitama 338-0832, Japan; ijichi@combi.co.jp

**Keywords:** heat-killed EC-12, functional foods, Min mice, intestinal polyps, colorectal cancer chemoprevention

## Abstract

Establishing effective methods for preventing colorectal cancer by so-called “functional foods” is important because the global burden of colorectal cancer is increasing. *Enterococcus faecalis* strain EC-12 (EC-12), which belongs to the family of lactic acid bacteria, has been shown to exert pleiotropic effects, such as anti-allergy and anti-infectious effects, on mammalian cells. In the present study, we aimed to evaluate the preventive effects of heat-killed EC-12 on intestinal carcinogenesis. We fed 5-week-old male and female *Apc* mutant Min mice diets containing 50 or 100 ppm heat-killed EC-12 for 8 weeks. In the 50 ppm treated group, there was 4.3% decrease in the number of polyps in males vs. 30.9% in females, and significant reduction was only achieved in the proximal small intestine of female mice. A similar reduction was observed in the 100 ppm treated group. Moreover, heat-killed EC-12 tended to reduce the levels of c-Myc and cyclin D1 mRNA expression in intestinal polyps. Next, we confirmed that heat-killed EC-12 suppressed the transcriptional activity of the T-cell factor/lymphoid enhancer factor, a transcriptional factor involved in cyclin D1 mRNA expression in intestinal polyps. Our results suggest that heat-killed EC-12 very weakly suppresses intestinal polyp development in Min mice, in part by attenuating β-catenin signaling, and this implies that heat-killed EC-12 could be used as a “functional food”.

## 1. Introduction

Colorectal cancer (CRC) is the most common cancer and a major cause of cancer-related deaths in advanced countries, including Japan. Moreover, the global burden of CRC is estimated to increase by 60% to more than 2.2 million new cases and 1.1 million deaths by 2030 [1]. Thus, establishing useful methods to prevent CRC is important. Fortunately, the development of sporadic CRC from normal mucosa takes an average of 10–20 years, thereby allowing us an opportunity for prevention. Lifestyle modifications, i.e., regular physical activity, smoking abstinence, and healthy nutrition, along with population screening methods for CRC check-ups, i.e., fecal occult blood testing and endoscopy, are popular CRC prevention methods. However, the change in lifestyle depends on personal intention, and the efficacy of such surveillance strategies is suboptimal and limits real effectiveness. Thus, we need to consider alternative preventative strategies, such as cancer chemoprevention, including the use of so-called “functional foods”.

Environmental factors, such as an excessive intake of lean meat, processed meat and alcohol, are known to potentially change the balance of intestinal bacteria and may also increase the risk of CRC [2]. On the other hand, the intake of dietary fiber, which is an important part in the nutrition of lactic acid bacteria (LAB), probably protects against CRC [2]. Dairy products and LAB have long been considered a critical part of healthy nutrition and have favorable effects on the colorectum. The anti-allergy and anti-infectious effects of LAB have been demonstrated in both human and in vivo models [3,4,5,6]. Recent studies have suggested that not only live LAB but also heat-killed LAB possess several beneficial effects [7,8,9,10,11]. Heat-killed LAB is suggested to possess immunomodulation function(s) without altering the intestinal microbiota.

*Enterococcus faecalis* strain EC-12 (EC-12) is a gram-positive bacterium that belongs to the LAB family. Its cell walls are reported to induce B-cell activation along with stimulation of IgA secretion in the intestine [12], which could remove pathogens from the intestine [13]. To date, several functions of EC-12 have been reported [14,15,16]. However, the preventive effects of heat-killed EC-12 on intestinal carcinogenesis have not yet been elucidated.

In this study, we demonstrated that administration of heat-killed EC-12 weakly decreased intestinal tumorigenesis in Min mice, *Apc*-mutant mice that develop many intestinal polyps through activation of β-catenin signaling. Moreover, we revealed that heat-killed EC-12 possesses a suppressive function of β-catenin signaling in vitro by measuring T-cell factor/lymphoid enhancer factor (TCF/LEF) transcriptional activity.

## 2. Results

### 2.1. Suppression of Intestinal Polyp Formation in Min Mice by Heat-Killed EC-12

Administration of 50 and 100 ppm heat-killed EC-12 to Min mice for eight weeks did not affect body weight, food intake or clinical symptoms, such as the appearance of the hair coat and movement activity throughout the experimental period (Figures S1 and S2). Final body weights (mean ± SD) for males were 25.7 ± 2.1 g (0 ppm treated control group) vs. 25.3 ± 0.9 g (50 ppm treated group); 26.1 ± 2.4 g (0 ppm) vs. 25.4 ± 1.0 g (100 ppm). Final body weights for females were 21.2 ± 1.2 g (0 ppm) vs. 20.7 ± 2.5 g (50 ppm); 21.1 ± 1.3 g (0 ppm) vs. 20.5 ± 2.6 g (100 ppm). There was no difference in the average daily food intake between each group of Min mice. The amount of food intake (mean ± SD) for males was 3.0 ± 0.3 g (0 ppm) vs. 2.9 ± 0.2 g (50 ppm); 3.3 ± 0.4 g (0 ppm) vs. 3.3 ± 0.3 g (100 ppm). The amount of food intake for females was 3.0 ± 0.5 g (0 ppm) vs. 3.0 ± 0.3 g (50 ppm); 3.2 ± 0.2 g (0 ppm) vs. 3.4 ± 0.3 g (100 ppm). There was no difference in the average daily food intake between each group of Min mice. In addition, no changes in major organ weights or the macroscopic view of organs that may have been indicative of toxicity were observed at the end of the experiment. These organs included the liver and kidneys. Table 1 and Table 2 summarize the data regarding the number and distribution of intestinal polyps in the basal diet control group and the 50 or 100 ppm heat-killed EC-12-treated group. In the two independent experiments, the majority of polyps developed in the small intestine, while only a few developed in the colon.

As shown in Table 1, there was a 4.3% decrease in the number of polyps in males vs. 30.9% in females in the 50 ppm treated group. A significant reduction in the number of polyps in females by 54.8% from the untreated control value was observed in the proximal segment of the small intestine (*p* < 0.05 compared to control group). As shown in Table 2, there was a 14.0% decrease in the number of polyps in males vs. 29.6% in females in the 50 ppm treated group. A significant reduction in the number of polyps in males by 25.8% from the untreated control value was observed in the middle segment of the small intestine (*p* < 0.05 compared to control group). This time, the proximal segment of the small intestine in the female group did not show a significant reduction in the number of polyps: 58.5% of the untreated control value. No significant differences in the numbers of polyps were observed in the other segments of the small intestine or the colon following heat-killed EC-12 treatment. Table 3 and Table 4 show the size distributions of the intestinal polyps in the basal diet and heat-killed EC-12-treated groups. The majority of polyps were approximately less than 3.0 mm in diameter. Heat-killed EC-12 treatment significantly reduced the number of small polyps in female mice (Table 3 and Table 4).

### 2.2. Weak Suppression of Gene Expression Regulated by β-Catenin Signaling in the Intestinal Polyps of Min Mice by Heat-Killed EC-12

**Figure 1:**
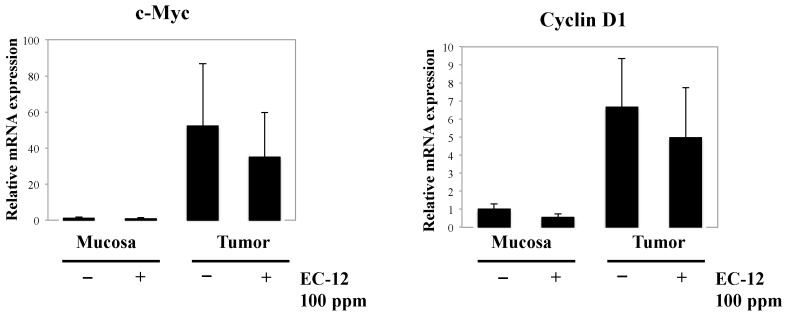
Effect of EC-12 (100 ppm) on the mRNA levels of cell proliferation-related factors in the intestines of Min mice. Quantitative real-time PCR anlysis was performed to determine c-Myc and cyclin D1 expression levels in the non-polyp (mucosa) and polyp portions of the intestines of female Min mice. Data were normalized to GAPDH. Each expression level in the non-polyp portions of the intestines in the control group (0 ppm) was set to one. Data are the mean ± SD, *n* = 5.

To clarify the mechanisms underlying heat-killed EC-12-mediated suppression of intestinal polyp formation/cell proliferation, gene expression that is regulated by β-catenin signaling in the non-polyp (mucosa) and polyp portions of the intestine was investigated in female mice (Figure 1). Real-time polymerase chain reaction (PCR) revealed that treatment with 100 ppm heat-killed EC-12 for eight weeks weakly but not significantly suppressed c-Myc and cyclin D1 mRNA expression in the intestinal polyp segments by 38% and 28% of the untreated control values, respectively. In the non-polyp portion, a similar weak decrease in cyclin D1 mRNA expression was observed between the heat-killed EC-12 treatment and non-treatment group. 

### 2.3. Suppression of TCF/LEF Promoter Transcriptional Activity by Heat-Killed EC-12

**Figure 2:**
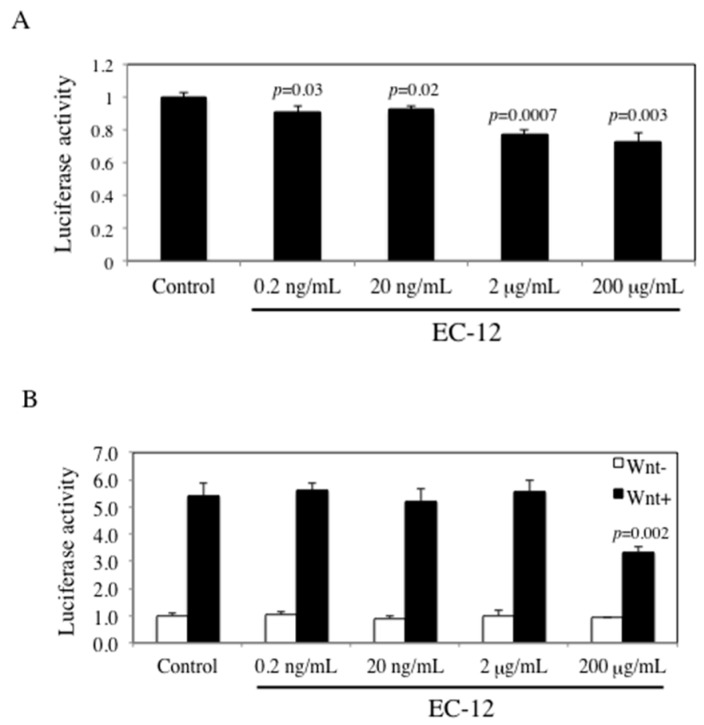
Effect of EC-12 on T-cell factor/lymphoid enhancer factor (TCF/LEF) promoter transcriptional activity in HCT116 cells. HCT116-TCF/LEF-Luc cells (**A**) and RKO-TCF/LEF-Luc cells (**B**) were treated with heat-killed EC-12 for 24 h. Wnt3a-conditioned medium was added 30 min after EC-12 treatment of RKO cells. The basal luciferase activity level of the control was set to 1.0. Data are the mean ± SD (*n* = 4).

To examine the effects of heat-killed EC-12 on β-catenin signaling, TCF/LEF promoter transcriptional activity was examined using a reporter gene assay following 24 h of heat-killed EC-12 treatment (0.2, 20 ng/mL, and 2, 200 µg/mL) in human colon cancer cells HCT116 and RKO. Heat-killed EC-12 treatment slightly decreased TCF/LEF promoter transcriptional activity in a dose-dependent manner in HCT116 cells (Figure 2A). Twenty-four hours of 200 µg/mL heat-killed EC-12 treatment decreased TCF/LEF promoter transcriptional activity by 22% (*p* < 0.01) of the untreated control value. RKO cells, which have intact *APC* and *β-catenin*, showed five-times higher TCF/LEF transcriptional activity by Wnt3a stimulation. Interestingly, 200 µg/mL heat-killed EC-12 suppressed TCF/LEF transcriptional activity by 38% (*p* < 0.01) of the untreated control value only with Wnt3a stimulation (Figure 2B).

### 2.4. No Obvious Changes Were Observed in the Enterobacterium with and without Heat-Killed EC-12 Treatment

**Figure 3:**
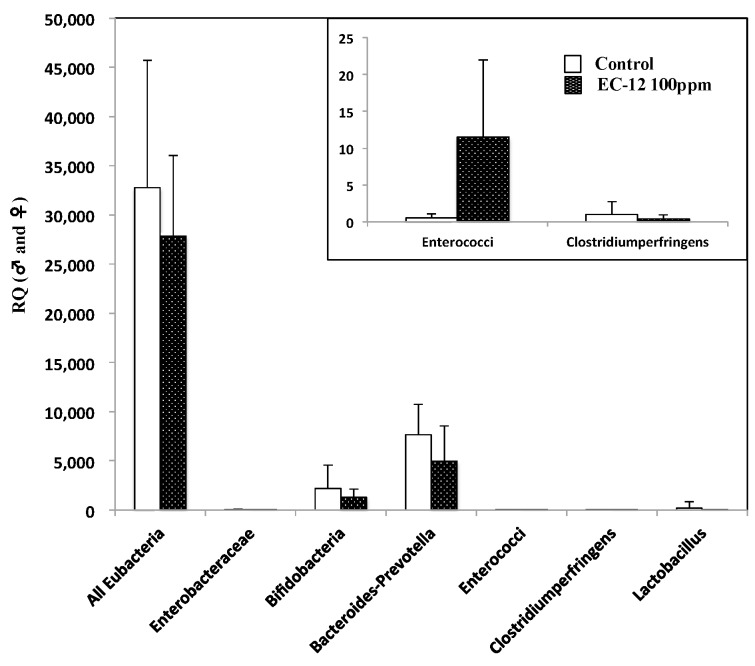
Effect of EC-12 on enterobacterium in the feces of Min mice. Feces of five mice in each group were collected from the rectum of Min mice. The amount of the indicated bacterium was evaluated by quantitative real-time PCR analysis. The window shows an enlarged view of the Figure, especially the amount of *Enterococci* and the *Clostridium perfringens* group. Open column: control group; closed column: 100 ppm EC-12-treated group. Data are the mean ± SD (*n* = 5). RQ: relative quantification.

To examine the effects of heat-killed EC-12 on the amount of enterobacterium, the total amount of enterobacterium, such as *Enterobacteraceae*, *Bifidobacteria*, *Bacteroides-Prevotella* group, *Enterococci*, *Clostridium* perfringens group, and Lactobacilli, were evaluated (Figure 3). As expected, no obvious changes were observed with heat-killed EC-12 treatment except for in the amount of Enterococci, in which heat-killed EC-12 in the diet resulted in its detection.

## 3. Discussion

In the present study, we assessed the effectiveness of heat-killed EC-12 as a “functional food” for CRC prevention by evaluating its ability to suppress intestinal polyp development in Min mice. We found that heat-killed EC-12 very weakly inhibited intestinal polyp development in the mice. We also confirmed that the expression levels of the downstream targets of β-catenin signaling, such as cyclin D1 and c-Myc, tended to be decreased in the polyp portions of the intestine. We finally confirmed that heat-killed EC-12 suppressed β-catenin signaling in an in vitro system.

The first in vivo study that provided evidence that lyophilized LAB acts against colorectal carcinogenesis showed that dietary administration of lyophilized cultures of *Bifidobacterium longum*, a lactic acid-producing enterobacterium, significantly suppressed the development of azoxymethane (AOM)-induced male F344 aberrant crypt foci, putative premalignant lesions, in the rat colon [17], and the second study demonstrated its action against tumors [18]. The results also revealed that ingestion of *B. longum* significantly inhibited AOM-induced ornithine decarboxylase (ODC, EC 4.1.1.17) activity and expression of p21. To our knowledge, this is the first study to provide evidence that ingestion of heat-killed EC-12 strain, a lactic acid-producing bacterium present in the human colon, inhibits intestinal polyp development in Min mice. Of note, reduction of small polyps in the female Min mice suggested that EC-12 could affect the very early stage of polyp development. Regarding the gender dependent response observed in this study, it has been reported that male Min mice develop more intestinal polyps than females do [19]. It is suggested that estrogen decreases peroxisome proliferator-activated receptor (PPAR) γ expression in mice [20]. Thus, it is interesting to examine the effects of EC-12 on PPARγ expression in future experiments to explain the gender dependent response.

The mechanism of suppressing intestinal tumor development by heat-killed EC-12 is not clear. On the other hand, it is well-known that germline mutations in the *APC* gene cause Familial adenomatous polyposis. In this hereditary cancer syndrome, loss of APC function leads to the inappropriate stabilization of β-catenin and the formation of constitutive complexes with the TCF family, leading to the expression of downstream genes that result in the development of intestinal polyps [21]. The downstream genes involve cyclin D1 and c-Myc, which also could be used as markers of proliferation, and these molecules play an important role in cell proliferation and anti-apoptotic response. In our experiment, *Apc*-mutant Min mice showed low expression of cyclin D1 and c-Myc in the intestinal polyp segments, implying the involvement of β-catenin signaling suppression in the mechanism. Thus, we confirmed that β-catenin signaling was suppressed by heat-killed EC-12, as shown in the in vitro study (Figure 2). To our knowledge, this is the first study to provide evidence for the interaction between LAB and β-catenin signaling. Finding a more potent LAB strain that suppresses β-catenin signaling might be desirable in the future.

Heat-killed EC-12 treatment tends to reduce polyp development in the small intestine but only significantly in the proximal to middle portion of the small intestine. This data could also be a clue to determine the mechanism behind heat-killed EC-12 treatment. LPL inducers NO-1886 and PPAR ligands have been shown to significantly reduce the number of intestinal polyps in the proximal part of the intestine [22,23]. On the other hand, indomethacin, a cyclooxygenase (COX) inhibitor; nimesulide, a COX-2-selective inhibitor; sesamol, a COX-2 suppressor; and apocynin, an NADPH oxidase inhibitor, have been shown to mainly reduce the number of intestinal polyps in the middle to distal parts of the small intestine [24,25,26,27]. We surmised that lipid–related metabolism plays a role in the effects of heat-killed EC-12 treatment on proximal intestinal polyp development. However, the effect of heat-killed EC-12 on lipid–related metabolism is not well known. Only an improvement in intestinal villous atrophy, which plays a pivotal role in lipid assimilation by heat-killed EC-12, has been reported [28]. To determine the suppressive mechanism of heat-killed EC-12, further examination is required.

In this study, we use 50 ppm and 100 ppm, equivalent to ~0.5 and 0.9 g EC-12/day intake for humans. We assumed that around 1 g/day EC-12 might be able to be consumed by humans. The heat-killed EC-12 content in ten cups of yogurt containing 10^11^ bacteria in each cup is equivalent to 20 mg of heat-killed EC-12. Moreover, heat-killed EC-12 is able to be included in processed food. Thus, the advantage of using heat-killed EC-12 as a “functional food” is that we do not have to worry about the intake amount, in other words, the effective dose of EC-12. Moreover, agents targeting β-catenin signaling might be useful for the prevention of other cancers, such as hepatocellular carcinomas and melanoma.

Our study has some limitations. In the Min mice model, it is known that only intestinal adenomas are developed before the age of 20 weeks. We only observed a very modest modulation of the polyp development, and have not examined the effects of EC-12 on adenocarcinoma formation. Further experiments using an experimental model of AOM/dextran sodium sulfate (DSS), for instance, are needed to provide more evidence for heat-killed EC-12 as a functional food.

In conclusion, this study demonstrated that heat-killed EC-12 very weakly suppresses the development of intestinal polyps in Min mice. Our findings imply that heat-killed EC-12 is a useful “functional food” for cancer prevention.

## 4. Materials and Methods

### 4.1. Chemicals

EC-12, a commercial product of the cell preparation of *E. faecalis* strain EC-12 (International Patent Organism Depositary in Japan number, FERM BP-10284; GenBank Accession number, AB154827; Combi Corp., Saitama, Japan) was used. This is a dried powder of heat-killed bacterium.

### 4.2. Cell Culture

HCT116 and RKO cells, human colon adenocarcinoma cells, were purchased from the American Type Culture Collection (Manassas, VA, USA). Both cell lines cells were maintained in DMEM supplemented with 10% heat-inactivated fetal bovine serum (FBS; HyClone Laboratories Inc., Logan, UT, USA) and antibiotics (100 µg/mL streptomycin and 100 U/mL penicillin) at 37 °C with 5% CO_2_.

### 4.3. Animals

Male and female C57BL/6-*Apc^Min/^*^+^ mice (Min mice) were purchased from Jackson Laboratory (Bar Harbor, ME, USA). The mice (*n* = 3–4) were housed in plastic cages with sterilized softwood chips as bedding in a barrier-sustained animal room maintained at 24 ± 2 °C and 55% humidity under a 12-h light/dark cycle. Heat-killed EC-12 was mixed with an AIN-76A powdered basal diet (CLEA Japan, Inc., Tokyo, Japan) at concentrations of 50 and 100 ppm.

### 4.4. Animal Experimental Protocol

Seven to ten male and female Min mice aged 5 weeks were given 50 or 100 ppm EC-12 for 8 weeks. All animals housed in the same cage were included in the same treatment group. Food and water were available ad libitum. The animals were observed daily for clinical symptoms and mortality. Body weight and food consumption were measured weekly. At the sacrifice time point, the mice were anesthetized with isoflurane, and blood samples were collected from their abdominal veins. Their intestinal tracts were removed and separated into the small intestine, cecum and colon. The small intestine was divided into a proximal segment (4 cm in length), and the rest of the segment was divided in half, containing the middle and distal segments. The number of polyps in the proximal segment was counted and collected under a stereoscopic microscope. The remaining intestinal mucosa (non-polyp portion) was removed by scraping, and the specimens were stored at −80 °C until quantitative real-time PCR analysis. The other regions were opened longitudinally and fixed flat between sheets of filter paper in 10% buffered formalin. Polyp numbers, size and intestinal distributions were assessed with a stereoscopic microscope. All experiments were performed according to the “Guidelines for Animal Experiments in the National Cancer Center” and were approved by the Institutional Ethics Review Committee for Animal Experimentation of the National Cancer Center (permission code: T05-022-C11, approval date: 1 April 2014). The animal protocol was designed to minimize pain or discomfort to the animals. The animals were acclimatized to laboratory conditions for more than two weeks prior to experimentation.

### 4.5. Bacterial DNA Extraction from Feces

Feces were collected from the rectum when each mouse was sacrificed. Samples not analyzed immediately were stored at −20 °C. To each sample, 0.3 g glass beads (0.5 mm diameter), 500 µL TE-phenol, and 300 µL breaking solution (2% Triton X-100, 1% SDS, 100 mM NaCl, 10 mM Tris-HCl (pH 8.0), 1 mM EDTA) were added for DNA extraction. The sample was ground for 30 s using an MM300 apparatus (Retsch, Haan, Germany) twice. The supernatant was transferred to a new microcentrifuge tube after centrifugation for 5 min at 14,000 rpm. An equal volume of phenol-chloroform-isoamyl alcohol was added to the supernatant, mixed well, and then centrifuged at 14,000 rpm for 5 min. Genomic DNA was precipitated using isopropanol, and after precipitation, the pellet was washed with 70% ethanol solution. The pellet was air-dried at room temperature and then dissolved into a suitable volume of DNA hydration buffer (Qiagen, Hilden, Germany).

### 4.6. Luciferase Assays for TCF/LEF Promoter Transcriptional Activity in Stable Transfectants

To measure TCF/LEF transcriptional activity, HCT116 and RKO colon cancer cells were transfected with TCF/LEF-Luc (Promega, Madison, WI, USA) reporter plasmids using Polyethylenimine MAX MW 40,000 (PolySciences, Warrington, PA, USA). The transfected cells were cultured for an additional 24 h. Cells stably expressing TCF/LEF-Luc were treated with hygromycin and cloned. These cells were referred to as HCT116-TCF/LEF-Luc and RKO-TCF/LEF-Luc cells, respectively. HCT116-TCF/LEF-Luc cells were seeded in 96-well plates (2 × 10^4^ cells/well). After a 24 h incubation, the cells were treated with EC-12 for 24 h. Wnt3a-conditioned medium was added 30 min after EC-12 treatment of RKO cells. Luciferase activity levels were determined using the Bright GLO Luciferase Assay System (Promega). Basal TCF/LEF luciferase activity in the control was set to 1.0. Data are expressed as the mean ± SD (*n* = 4).

### 4.7. Quantitative Real-Time PCR Analysis for Tissue Sample Evaluation

Tissue samples from the proximal segment of small intestinal mucosa and polyps of mice were rapidly deep-frozen in liquid nitrogen and stored at −80 °C.

Total RNA was isolated from tissue samples by using RNAiso Plus (TaKaRa, Shiga, Japan); 100 ng aliquots in a final volume of 20 µL were used for synthesis of cDNA using a High Capacity cDNA Reverse Transcription Kit (Applied Biosystems, Foster City, CA, USA) and oligo (dT) primers. Real-time PCR was carried out using the CFX96/384 PCR Detection System (BIO RAD, Tokyo, Japan) and Fast Start Universal SYBR Green Mix (Roche Diagnostics, Mannheim, Germany), according to the manufacturers’ instructions. The primer sequences were as follows: c-Myc (5′-GCTCGCCCAAATCCTGTACCT and 3′-TCTCCACAGACACCACATCAATTTC), cyclin D1 (5′-TGACTGCCGAGAAGTTGTGC and 3′-CTCATCCGCCTCTGGCATT) and GAPDH (5′-TTGTCTCCTGCGACTTCA and 3′-CACCACCCTGTTGCTGTA). To assess the specificity of each primer set, the melting curves of the amplicons generated by the PCR reactions were analyzed.

### 4.8. Quantitative Real-Time PCR Analysis for Enterobacterium Evaluation

As a DNA template, 40 ng of DNA in each sample was used for real-time PCR. PCR reactions were performed in a 7900HT Fast Real-Time system, with Fast SYBR^TM^ Green PCR Master Mix (Thermo Fisher Scientific, Vilnius, Lithuania). The primer sequences were as follows: All eubacteria (5′-ACTCCTACGGGAGGCAGCAGT and 5′-GTATTACCGCGGCTGCTGGCAC), Enterobacteraceae (5′-CATTGACGTTACCCGCAGAAGAAGC and 5′-CTCTACGAGACTCAAGCTTGC), Bifidobacteria (5′-TCGCGTC(C/T)GGTGTGAAAG and 5′-CCACATCCAGC(A/G)TCCAC), Bacteroides-Prevotella group (5′-GGTGTCGGCTTAAGTGCCAT and 5′-CGGA(C/T)GTAAGGGCCGTGC), Enterococci (5′-CCCTTATTGTTAGTTGCCATCATT and 5′-ACTCGTTGTACTTCCCATTGT), Clostridium perfringens group (5′-ATGCAAGTCGAGCGA(G/T)G and 5′-TATGCGGTATTAATCT(C/T)CCTTT), and all lactobacilli (5′-TGGAAACAG(A/G)TGCTAATACCG and 5′-GTCCATTGTGGAAGATTCCC) [29,30]. Wells included 10 µL of 2× SYBR Green master mix, 6.4 µL of distilled water, 2 µL of the DNA sample (20 ng/µL) and 0.8 µL of primer set (5 µM each) for a total of 20 µL in each well. Plates were run at 95 °C for 20 s, and then 40 cycles of 95 °C for 1 s and 60 °C for 20 s. Samples were run in quadruplicate.

### 4.9. Statistical Analyses

All results are expressed as the mean ± SD, and all statistical analyses were performed using Student’s *t*-tests, except for the luciferase assay. The luciferase assay was analyzed using Dunnett’s test. Differences were considered statistically significant at * *p* < 0.05.

## Figures and Tables

**Table 1:** Effect of *Enterococcus faecalis* strain EC-12 (EC-12) (50 ppm) on the number of polyps in Min mice.

Dose (ppm)	Gender (Number of Mice)	Small Intestine			Colon	Total
		Proximal	Middle	Distal		
0	Male (8)	6.3 ± 4.3	9.3 ± 3.5	29.3 ± 7.6	1.8 ± 2.0	46.5 ± 11.6
50	Male (10)	6.7 ± 3.1	11.8 ± 6.4	25.7 ± 11.7	0.3 ± 0.7	44.5 ± 18.1
0	Female (9)	8.4 ± 5.2	12.2 ± 6.7	28.6 ± 15.5	0.1 ± 0.3	48.2 ± 19.7
50	Female (8)	3.8 ± 2.0 *	10.0 ± 3.2	18.1 ± 11.3	0.8 ± 0.7	33.3 ± 13.9

Data are presented as the means ± SD. Significantly different from the untreated control group at * *p* < 0.05.

**Table 2:** Effect of EC-12 (100 ppm) on the number of polyps in Min mice.

Dose (ppm)	Gender (Number of Mice)	Small Intestine			Colon	Total
		Proximal	Middle	Distal		
0	Male (9)	5.3 ± 2.1	9.3 ± 3.2	23.1 ± 2.9	1.4 ± 2.0	39.2 ± 4.2
100	Male (9)	2.3 ± 0.9	6.9 ± 2.9 *	23.7 ± 8.3	0.8 ± 1.6	33.7 ± 11.7
0	Female (8)	5.3 ± 2.6	9.4 ± 2.8	25.9 ± 11.1	1.8 ± 3.5	42.3 ± 10.3
100	Female (8)	3.1 ± 2.0	8.5 ± 2.8	17.8 ± 9.3	0.4 ± 0.5	29.8 ± 13.0

Data are presented as the means ± SD. Significantly different from the untreated control group at * *p* < 0.05.

**Table 3:** Effect of EC-12 (50 ppm) on the size distribution of intestinal polyps in Min mice.

Dose (ppm)	Gender	Diameter (mm)									
		<0.5	0.5 to <1.0	1.0 to <1.5	1.5 to <2.0	2.0 to <2.5	2.5 to <3.0	3.0 to <3.5	3.5 to <4.0	4.0 to <4.5	≥4.5
0	Male	13.4 ± 6.5	19.6 ± 7.1	7.0 ± 3.0	3.6 ± 2.9	2.0 ± 2.1	0.3 ± 0.5	0.3 ± 0.5	0.1 ± 0.4	0.3 ± 0.5	0.0 ± 0.0
50	Male	12.2 ± 6.1	21.2 ± 10.1	7.0 ± 6.1	3.1 ± 2.4	0.5 ± 0.7	0.2 ± 0.4	0.2 ± 0.4	0.0 ± 0.0	0.0 ± 0.0	0.03 ± 0.2
0	Female	15.8 ± 7.2	23.7 ± 12.4	6.1 ± 4.7	1.6 ± 0.9	0.9 ± 0.8	0.1 ± 0.3	0.1 ± 0.3	0.0 ± 0.0	0.0 ± 0.0	0.0 ± 0.0
50	Female	8.3 ± 4.3 *	14.9 ± 9.1	5.5 ± 2.1	3.3 ± 3.1	0.9 ± 0.6	0.1 ± 0.4	0.3 ± 0.5	0.0 ± 0.0	0.1 ± 0.4	0.0 ± 0.0

Data are presented as the means ± SD. Significantly different from the untreated control group at * *p* < 0.05.

**Table 4:** Effect of EC-12 (100 ppm) on the size distribution of intestinal polyps in Min mice.

Dose (ppm)	Gender	Diameter (mm)									
		<0.5	0.5 to <1.0	1.0 to <1.5	1.5 to <2.0	2.0 to <2.5	2.5 to <3.0	3.0 to <3.5	3.5 to <4.0	4.0 to <4.5	≥4.5
0	Male	7.2 ± 4.4	16.6 ± 2.8	8.1 ± 3.6	4.3 ± 1.8	2.1 ± 0.8	0.8 ± 0.8	0.0 ± 0.0	0.0 ± 0.0	0.0 ± 0.0	0.1 ± 0.3
100	Male	3.9 ± 4.2	12.9 ± 4.5	10.4 ± 4.9	4.6 ± 2.0	1.4 ± 1.0	0.1 ± 0.3	0.2 ± 0.4	0.1 ± 0.3	0.0 ± 0.0	0.0 ± 0.0
0	Female	9.9 ± 4.3	20.9 ± 7.2	6.1 ± 3.0	2.5 ± 1.1	1.8 ± 1.4	0.6 ± 0.7	0.3 ± 0.5	0.0 ± 0.0	0.3 ± 0.5	0.0 ± 0.0
100	Female	3.9 ± 3.6 *	12.5 ± 7.6 *	7.9 ± 5.1	3.9 ± 2.0	1.1 ± 1.0	0.4 ± 0.5	0.0 ± 0.0	0.0 ± 0.0	0.1 ± 0.4	0.0 ± 0.0

Data are presented as the means ± SD. Significantly different from the untreated control group at * *p* < 0.05.

## Supplementary Materials

Supplementary materials can be found at www.mdpi.com/1422-0067/18/4/826/s1.

## Abbreviations

AOM	Azoxymethane
COX	Cyclooxygenase
CRC	Colorectal cancer
DSS	Dextran sodium sulfate
EC-12	*Enterococcus faecalis* strain EC-12
FBS	Fetal bovine serum
GAPDH	Glyceraldehyde-3-phosphate dehydrogenase
LAB	Lactic acid bacteria
PCR	Polymerase chain reaction
PPAR	Peroxisome proliferator-activated receptor
