# Supplementary material for: Preventive Effects of Heat-Killed Enterococcus faecalis Strain EC-12 on Mouse Intestinal Tumor Development
Source: Int J Mol Sci. 2017 Apr 13;18(4):826. doi: 10.3390/ijms18040826 (PMC5412410; doi:10.3390/ijms18040826)
Supplement: Supplementary file 1 [file ijms-18-00826-s001.pdf]

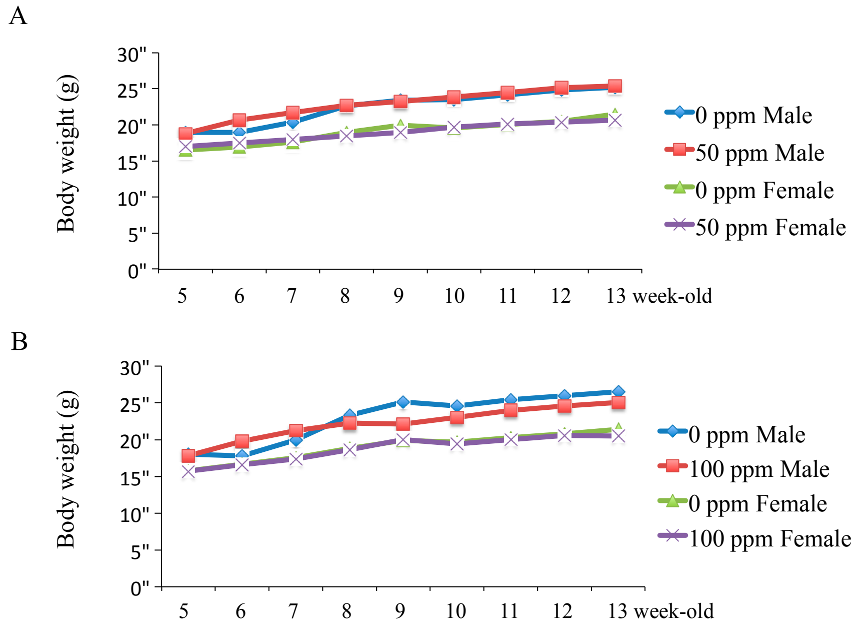

Figure S1. Body weight change during EC-12 administration in Min mice.

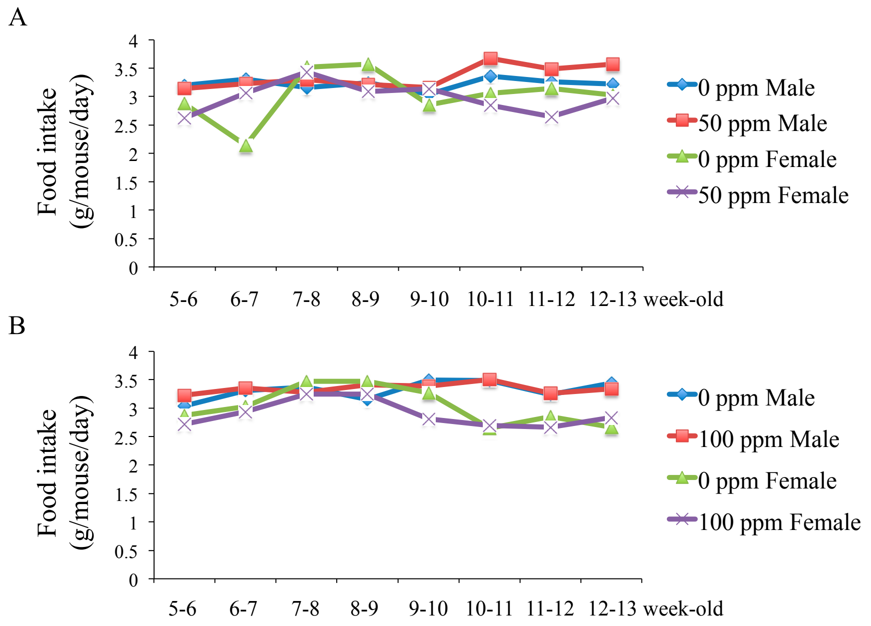

Figure S2. Food intake amount of mouse per day in the experimental period.
